# Sero-prevalence and risk factors of brucellosis among suspected febrile patients attending a referral hospital in southern Saudi Arabia (2014–2018)

**DOI:** 10.1186/s12879-020-4763-z

**Published:** 2020-01-09

**Authors:** Abdullah M. Alkahtani, Mohammed M. Assiry, Harish C. Chandramoorthy, Ahmed M. Al-Hakami, Mohamed E. Hamid

**Affiliations:** 1 Department of Microbiology and Clinical Parasitology, College of Medicine, King Khalid University, PO Box 641, Abha, 61421 Saudi Arabia; 2 Main laboratory, Aseer Central hospital, Ministry of Health, Abha, Saudi Arabia

**Keywords:** Public health, Zoonotic disease, Slide agglutination test, Risk factors

## Abstract

**Background:**

Human brucellosis is an infectious zoonotic disease caused by *Brucella* spp. It is one of the most public health problems that remains largely neglected in developing counties, including Saudi Arabia. Brucellosis is particularly prevalent among rural people who have constant contact with livestock.

**Methods:**

A cross-sectional sero-epidemiological study conducted in Aseer Central Hospital, South Saudi Arabia, between 2014 and 2018 among 7567 patients. Serum samples were analyzed for *Brucella* antibodies using slide agglutination test. Serology results and patient’s demographic data were analyzed by GraphPad Prism. Results were presented as mean ± SEM and differences between two groups were assessed by t-test and *p* < 0.05 was considered significant.

**Results:**

The prevalence of brucellosis among the admitted suspected 7567 cases was 12.8% (10.4–15.7%; CI 95%). The highest prevalence rate was detected during 2015, the rate decreased to the lowest level during the last three years (*p* < 0.05). Higher rate of brucellosis was observed among males than females (p < 0.05) and most cases were reported during summer season (p < 0.05). The highest prevalence rate was observed in age group 21–40 year old (40.5%) followed by 41–60 years (27.7%). The lowest prevalence rate was noticed in old and young children (15 and 3%, respectively). Cross-transmission of brucellosis was seen within family (1%) and high titers (> 1280) was noticed in 22% of the hospitalized patients. The major symptoms were fatigue, hyperhidrosis, fever and joint pain.

**Conclusion:**

Our findings showed a high prevalence of human brucellosis among suspected patients in Aseer region. This indicates that clinical suspicion is a valid criterion and the endemic nature of the disease. The disease status requires early laboratory detection and confirmation to start prompt treatment to decrease patients suffering.

## Background

Mediterranean fever is an extremely infectious zoonotic disease caused by *Brucella* spp., which is a Gram-negative bacteria that affects humans and animals and poses a serious threat to public health. Presently, the genus consists of 11 nominal species, including*, suis* and *B. melitensis* which are the most significant source of disease in small ruminants and cattle, respectively [1]. These species are transmitted between animals both vertically and horizontally, causing abortion and infertility in their primary natural hosts—goats and sheep (*B. melitensis*), sows (*B. suis*), and cows (*B. abortus*) [2]. *Brucella* shows host favoritism, but is not host definite, and spillover can happen when different host species are kept together or share grazing grounds and water sources [3]. This disease is transmitted through contact with infected animals and humans, and the consumption of unpasteurized dairy and infected products. The probability of person-to-person transmission is unconfirmed, but likely, as it was described in Royal Oak, Michigan, in the United States, when the microorganisms were isolated in an infected microbiologist’s wife, demonstrating that the sexual contact could be a cause of infection [4]. Human brucellosis causes a flu-like sickness with fever, malaise, myalgia, weight loss, and weakness. Clinical diagnosis is interesting, and the disease is usually not easy to diagnose and may be misdiagnosed as malaria or other diseases associated with fevers. It is thought that for every case of brucellosis diagnosed, four cases are thought to go undetected [5]. *Brucella* is one of the causes of fever of an extended duration in endemic areas and an important cause of fever of an unknown origin (FUO) [6, 7].

More than half a million new cases of the disease are reported annually, with around 10 per 100,000 population [8]. Furthermore, brucellosis causes significant economic problems to the animal industry worldwide because it generally causes abortion, infertility, and a reduction in milk and meat production [9]. Brucellosis has been eradicated in many developed countries, but it is still endemic in several areas, especially in the Mediterranean region [10]; Africa [3, 11]; and some developed countries with a low income, limited resources, and frequent contact with livestock animals (sheep, goats, cattle, water buffalo, camels, and pigs) [12]. Its prevalence differs globally as a high incidence rate was reported in most African countries, being higher than in other countries worldwide. Furthermore, a high incidence rate was reported in the Aseer region of the Kingdom of Saudi Arabia (KSA) between 2004 and 2012 [13].

Slide agglutination test is used routinely to screening human and animal brucellosis in many countries which detects antibodies against brucella in serum. The Slide agglutination test is a rapid, comparatively low-cost and effective for the diagnosis of brucellosis. However, it may give false negative results since numerous factors influence its reaction and reading [14]. The main precaution that can be taken for the prevention of brucellosis infection is the elimination of raw meat and unpasteurized animal products, including milk and cheese, and the promotion of personal protection, such as using thick gloves, spectacles, and dresses for individuals who are in direct contact with animals. Vaccination is regulated for some animals, especially in the case of strains of *Brucella abortus* and *Brucella melitensis*. For humans, research is still in progress, and nothing has been confirmed [15, 16]. The treatment is achieved with a combination of doxycycline and rifampin antibiotics, for 42 days, in addition to an assessment of the clinical symptoms, especially for patients who are at a high risk, such as pregnant women and children [17].

The first case of human brucellosis in Saudi Arabia was documented at least35years ago [18]. Clinical characteristics of human brucellosis from the Riyadh area include back pain, subacute hepatitis, and arthritis, as well as abortion and endocarditis. In the Kingdom of Saudi Arabia, brucellosis is a wide spread infectious disease. The Ministry of Health (MOH) described a high incidence of brucellosis (18/100,000 population) in 2011 [13]. A number of studies from endemic areas have shown a high number of pediatric patients infected with this disease [19].

Another study has showed that brucellosis is a main health issue in the KSA [20]. Although the incidence of brucellosis decreased between the year 2004 and 2012, it was still higher than in other unindustrialized and industrialized countries [13, 21]. The prevalence of brucellosis among male citizens aged between 15 and 44 years represented the highest risk of acquiring brucellosis. It has been reported that Al-Qassim and Aseer in the South had the highest total of cases, followed by Hail and the Northern borders of Saudi Arabia [20]. Another investigation on the epidemiology of brucellosis was carried out among abattoir workers from slaughterhouse sites in different Saudi Arabian cities [22]. The frequency of brucellosis amongst workers was found to be 1.8%. Positive titers were obtained from veterinarians, butchers, and laborers; however, negative titers were obtained from administrative staff, drivers, and maintenance laborers.

Brucellosis has been an endemic disease in the KSA since the early 1980s. Many reasons for this have been explored, but the most obvious of them is the increased modernization that has occurred in the last forty years and the massive importation of animals from areas where brucellosis is endemic, such as different African countries. Housing camels is part of the KSA history, including the drinking of raw milk, eating of camel meat, and direct contact with infected animals or their products, which are the main routes of infection [23]. In addition, the consumption of fresh and unpasteurized camel’s milk is a traditional practice in different regions of KSA. In addition, KSA is the center of Islam, home to the Two Holy Mosques, the Haram Mosque and the Prophet’s Mosque, which are precious to every Muslim. Millions of pilgrims gather at these mosques to perform the Hajj rituals and Omrah.

The prevalence of brucellosis has been studied in KSA among different regions including our recent study among the agro-pastoral sectors in southern Saudi Arabia. The study determined certain areas and having animals (sheep) are risks of brucellosis [24]. This research extends the boundaries of these studies and includes the brucellosis distribution and determination from 2014 to 2018 in the Southern region of KSA, taking into account gender, age groups, and the seasonal distribution.

## Methods

### Ethical approval

Before starting data collection, ethical approval was obtained from the Research Ethics Committee of the College of Medicine, King Khalid University (#09-06-2018,) and after getting the permission of MOH to access the raw data. During the data collection stage, the information was anonymous, and confidentiality of data was assured.

### Study area

This present research was carried out in the Aseer region of Saudi Arabia, which has an estimated population of about 2.2 million inhabitants. It is located in the southwest of the Kingdom of Saudi Arabia and covers around 76,793 km^2^. It contains the country’s highest mountain, which rise to almost 3000 m (9800 ft) above the Red Sea.

### Study design and sampling procedure

This study was a cross-sectional sero-epidemiological study carried out at the Department of Microbiology, College of Medicine, King Khalid University and Aseer Central Hospital (ACH), Aseer region, Saudi Arabia, between 2014 and 2018, with the aim of determining the prevalence of brucellosis in the human population and determining risk factors related to the disease. Socio-demographic data (i.e., sex and age) of the patients were obtained from the hospital laboratory database. Data were collected over five years, involving the collection of samples from suspected patients following their consent. Gender is defined as female and male; age groups are identified as 1–20 years old, 21–40 years old, 41–60 years old, 61–80 years old, and 80–100 years old; and frequency of cases is expressed in months. Population data were used from 2014 to 2018 and classified by gender, age group, and incidence foreach month of every year. Data were collected from 7567 patients referred to ACH. Brucellosis data were collected from the ACH central laboratory, where cases are usually documented. Serological tests were used for the diagnosis of *Brucella* spp., followed by the determination of associated risk factors, such as age, sex, and season.

### Slide agglutination test and titration

A slide agglutination test was initially used to screen brucellosis. Serum samples were screened for febrile antibodies against *Brucella* spp. (*B. melitensis* and *B. abortus*) using the slide agglutination test obtained from Crescent Diagnostics (Crescent Diagnostics, Jeddah, Saudi Arabia) (Febrile antigens used were; FB850–10 *B. melitensis* and FB850–9 *B. abortus*). According to the manufacturer instructions, titers ≥1/180 indicate infection. A total of 50 *μ*l of each serum sample was placed on a clean glass slide and an equal volume of antigen was placed next to the sample to be tested. A sterile plastic stirrer was then used to mix the serum and the antigen thoroughly before slowly rocking the sample for 4 min to observe the agglutination. The result was appreciated by examining the degree of agglutination. Slightly notice able clumping was reported as positive; however, an absence of agglutination was considered as negative.

The titration of the *Brucella* antibodies was carried out by using a micropipette and the slide agglutination method. Different volumes (5, 10, 20, 40, and 80 *μ*l) of undiluted serum were placed in a separate circle of the slide test. Then, 50 *μ*l of *Brucella* antigen was added to each sample and the two were mixed together with a disposable stirrer and spread over the entire circle. The slide was placed on a mechanical rotator for 1 min. Then, the presence or absence of clumping was examined macroscopically. The presence of agglutination in the first, second, third, fourth or fifth well was reflected suggestive of a 1:20, 1:40, 1:80, 1:160 or 1:320 titer, respectively according to the manufacture instructions as shown in Table 1.

**Table 1:** The volumes added in each dilution and corresponding titration

Slide no.	1	2	3	4	5
Pt. Serum	5 μl	10 μl	20 μl	40 μl	80 μl
Brucella Ag	50 μl	50 μl	50 μl	50 μl	50 μl
Titer	1:20	1:40	1:80	1:160	1:320

The positive and negative controls were included to confirm the performance of this serological test. Standardized positive patient’s samples were used as a control in addition to control provided with the kit. Expected false negative samples were tested by tube method upon requested of treating physician.

### Statistical analysis

Data collected were illustrated and inserted into a Microsoft Excel 10 worksheet (Microsoft Corporation, Redmond, WA, USA). Expressive and investigative statistics were used to describe the data. Differences between the seroprevalence of brucellosis according to age, sex, and the seasonal incidence rate were assessed using GraphPad Prism version 8 for Windows (GraphPad Software, La Jolla California, USA). In all analyses, the confidence level was set at 95% and a *P* value < 0.05 was considered significant.

## Results

### Slide agglutination test for febrile brucellosis antibodies

A total of 7567sera samples were included in this study, and 975 (12.8%) were found to be positive for brucellosis when tested with the slide agglutination test*.* Titers ≥1/180 was taken to indicate positive infection. The results of positive brucellosis cases in relation to different epidemiological criteria are summarized in Table 2.

**Table 2:** “Data recorded from patients diagnosed with brucellosis in Aseer region between 2014 and 2018”

Data Year	2014	2015	2016	2017	2018
Number of samples	2047	1605	1346	1286	1283
Number of positive samples	258	253	166	164	134
% of positive samples	12.6	15.8	12.3	12.8	10.4
Brucellosis in males (%)	9.2	11.4	8.6	9.0	7.3
Brucellosis in females (%)	3.4	4.4	3.7	3.8	3.1
Brucellosis in age groups (%)					
0–20 yr.	11.2	11.5	24.1	12.2	15.7
21–40 yr.	44.3	45.3	36.1	38.4	35.8
41–60 yr.	30.2	28.1	24.1	32.9	23.1
61–80 yr.	12.4	11.9	13.3	16.5	18.7
81–100 yr.	1.9	3.2	2.4	0	6.7
Brucellosis in months (%)					
January	5	7	5	5	6
February	9	12	10	5	10
March	4	11	10	2	3
April	12	4	11	7	9
May	5	9	7	10	4
June	4	13	10	12	15
July	10	6	10	11	12
August	12	8	6	10	11
September	11	10	5	12	10
October	11	9	17	11	12
November	12	5	4	10	4
December	5	6	5	5	4

### Association of positive brucellosis according to years

A comparison of positive human brucellosis according to different epidemiological criteria (years, gender, age, and month) in the Aseer region between 2014 and 2018is shown in Table 2.

**Fig. 1:**
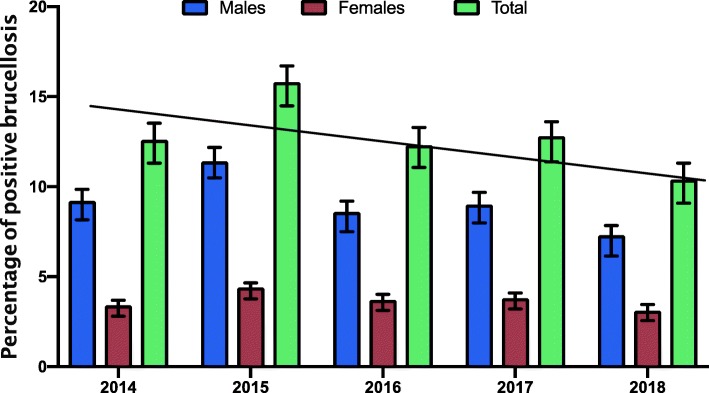
Seroprevalence of human brucellosis in males and female sin the Aseer region for five years. Error bars with standard errors are shown (I) and the line indicates the linear trend line

The highest prevalence rate was detected in 2015 (15.8%), followed by 12.8% in 2017 and 12.6% in 2014. The lowest percentage was reported in 2018 (10.4%). This high prevalence of human brucellosis was statistically significant when compared to the prevalence in the other years (*p* > 0.05) (Fig. 1).

### Association of positive brucellosis according to gender

The sex-specific seroprevalence showed a high incidence between males (7.3–11.4%) compared to the females (3.1–4.3%) in all years of this study (Table 2). There was no statistically significant association (*P* > 0.05) between sex and the presence of brucellosis in the Aseer region of Saudi Arabia. Regarding sex, there was no difference in the prevalence of human brucellosis in males and females over the years of this study. As shown in Fig. 1, the prevalence of the infection was two-thirds of the studied cases in males and one third in females. as Additionally, the frequency was constant during all the years.

### Association of positive brucellosis according to age

**Fig. 2:**
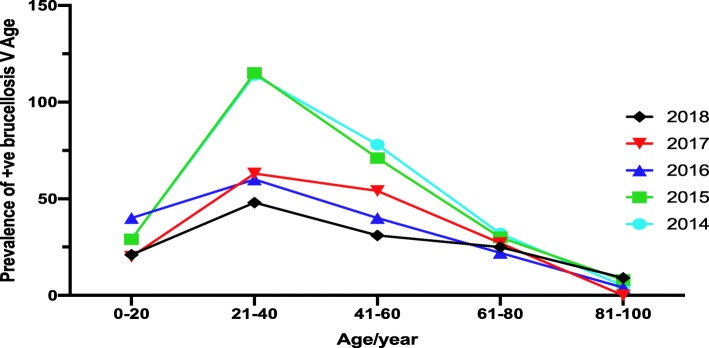
Distribution of positive human brucellosis among different age groups in the Aseer region for 5 years (2014*–*2018)

Regarding the age, the highest percentage of positive cases for brucellosis was in the age group of between 21 and 40 years old over the period of this study (35.8–45.3%), followed by the age group of between 41 and 60 years (23.1–32.9%) and the age group of between 61 and 80 years old (11.9–18.7%) (Table 2 and Fig. 2). The lowest rate was found in the age group of between 81 and 100 years, as shown in Fig. 2. There were no significant differences between the prevalence of this disease between 2014 and 2018 as the main age group is the same as who is at a high risk of infection.

There is a strong association between human brucellosis and age, as shown in Fig. 2. Significant differences between the prevalence of infection and age group of between 21 and 60 years among all years were noticed (2014–2018).

### Association of positive brucellosis according to season

**Fig. 3:**
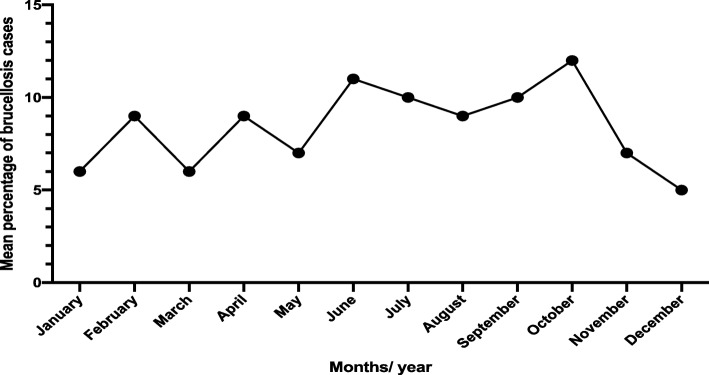
Reported cases of human brucellosis, by month of the year, in the Aseer region for 5 years (2014*–*2018)

According to this study, in 2014, 2017, and 2018, the highest incidences of human brucellosis were reported in summer (11–17%), while the highest rate in 2015 was detected in February (12%) and June (13%). On the other hand, the lowest frequency occurred in November and January (5–7%) over the period of this study (Fig. 3 and Table 2).

## Discussions

Brucellosis is the most public zoonotic infectious disease worldwide, affecting more than 500,000 people each year [25]. This study was conducted to determine the seroprevalence of human brucellosis in the Aseer region, southern Saudi Arabia, between 2014 and 2018, which follows up from the last study carried out between 2004 and 2012 [13]. as Additionally, the demographic profile of patients with brucellosis in terms of gender, age, and monthly incidence every year, were reported in this study. This study showed a high prevalence of human brucellosis of 12.8% over the period of this study in the suspected patients and the prevalence decreased in the last two years. The overall seroprevalence of the human brucellosis trend showed fluctuations, with a steady rate between 2014 and 2018 in the Aseer region. This can be compared with a study conducted between 2004 and 2012, which showed a high incidence rate (IR) of human brucellosis (27.1%) in Saudi citizens (the IR was calculated per 100,000 persons for the total population) [13]. Another study showed a low prevalence of brucellosis (2.6%) in the Al-Medina region, Kingdom of Saudi Arabia [26]. On the other hand, a very high prevalence of brucellosis (38.03%) was documented in Hawtat Sudair city, KSA [27]. As can be seen from these research studies, the prevalence of brucellosis in Saudi Arabia is not well-documented and the fact that it is different geographically from one region to another means the factors related to this infectious disease are very important and need extended studies. Moreover, the high prevalence of human brucellosis in the Aseer region is more likely to be caused by the lifestyle of most people in Saudi Arabia, which is a mixture of modern and traditional lifestyles [13].

In this study, around 71% of studied cases were males and 29% were females. The mean (± SD) of cases was about ±2.3. Our results are similar to those of a study carried out in Kiboga District, Central Uganda, in which the prevalence was higher in males (20.5%) than females [28], but the rate of brucellosis in females was high in Hamadan Province in Iran [29]. A study by Al-Eissa found that brucellosis presents in both genders of the Saudi population and that the most common way of getting brucellosis is through the consumption of untreated milk or milk products acquired mainly from infected livestock, which is a traditional practice that is upheld and represents the nutritional habits of the people [30]. Saudi males commonly have more occasions to drink raw milk than females. Men usually go camping for days in the countryside, and they like to drink fresh camel milk from local shepherds. Another reason for the high brucellosis prevalence in males could be that the disease is mainly a work-related infection (abattoirs and veterinarians) [31].

In this study, the population aged between 21 and 40 years was at a high risk of brucellosis, followed by the age group of between 41 and 60 years old. Numerous studies have shown the prevalence of brucellosis in different regions in Saudi Arabia using these age groups [13, 32]. These studies also approved that younger aged cases have a lower incidence of brucellosis, which is consistent with our observations and with other studies carried out in Kuwait [33]. These data are in parallel with our study where we showed that populations with an age of less than 20 years and older than 60 are less infected compared to those with an age from 21 to 60 years (Table 2 and Fig. 2). This is very likely because people are coming into contact with infected animals more often when they become adults. Moreover, it is most likely because children come into contact with infected animals less often than adults.

The seasonal distribution of positive human brucellosis (Fig. 3) showed that the highest number of brucellosis cases was observed in the summer season (11–17%), followed by the winter season. Studies indicated that warmer weather is the most suitable environment, permitting the survival of *Brucella* organisms and the transmission of this zoonotic disease [34]. It is clear from the results of this study that the seasonality of human brucellosis might be related to different risk factors, such as rainfall, sunshine, and the consumption of a large amount of milk and other products from infected animals during the summer, especially in the Aseer region.

Currently, brucellosis in Saudi Arabia is facing a series of challenges that include the necessary assessment of the prevalence of the disease in humans and animals. It also includes wildlife and the various local epidemiological features, with several important methodological diagnostic gaps that concern the tools used to detect, control, and eradicate this infectious disease. *Brucella* infection among people living in the countryside communities is an important public health problem in the Aseer region of Saudi Arabia [13]. Males living in rural areas who usually engage in the processing of raw milk and milk products are particularly at risk of *Brucella* infection. Age is a risk factor for *Brucella* infection in humans in the study area [35]. Human brucellosis cases increased during the summer season in the Aseer region [13] and more in the northwestern Aseer area and among those possessing animals notably sheep are at higher risk of brucellosis [24].

The limitation of this study is that including the serological slide agglutination test used in this project was not enough to distinguish the different *Brucella* spp. from each other. Secondly, the patient records in which there was extra information, such as job or history of contact with infected animals, represents a limitation. Thirdly, these data could not be connected to data from agriculture or veterinarian records. The association of human brucellosis data to the animal counterpart could be useful bases for disease control approaches. Therefore, the establishment of this linkage represents further research. The fourth limitation is that it was not possible to discriminate between recurrence and treatment failure.

Several limitations are present in our study; firstly, the slide agglutination tests may have a potential cross-reactivity with IgM of different organisms such as *Francisella tularensis, Salmonella urbana,* and some other bacteria. In addition, this test has low sensitivity and specificity especially in chronic disease and endemic areas. Confirmatory tests were not performed, and this might explain the quite high positive results in this test.

As a retrospective study, our research took advantage of the available risk factors and analyzed them; further studies should be designed to incorporate various factors that might have an effect on the disease occurrence.

## Conclusions

Our findings showed a high prevalence of human brucellosis among suspected patients in the Aseer region. This indicates that high clinical suspicion is a valid criterion, which will require early laboratory detection and confirmation to start prompt treatment to decrease patient suffering. Based on the results reported here and other concordant published evidence, we advise that concern should be given to a combined human-animal brucellosis control program in the studied region and that surveys aimed at assessing the frequency of ruminant brucellosis are carried out in other parts of the country.

Community alertness, particularly in countryside societies, to spread awareness about brucellosis and related factors which increase the risk should be developed. Drinking raw milk must mainly be dispirited. Organized approaches, such as the immunization, isolation, and eradication of diseased animals, as well as careful hygiene within the manufacturing process, should be applied with more awareness of the seasonality. Additional research is required to discover more about the causes associated with the seasonality.

## Data Availability

The datasets used in the current study are available from the corresponding author on reasonable request.

## Abbreviations

ACH: Aseer Central Hospital
FUO: Fever of unknown origin
IR: Incidence Rate
KSA: Kingdom of Saudi Arabia
MOH: Ministry of Health
SD: Standard Deviation
SEM: Standard Error Mean
